# Molecular Mechanisms of Vascular Tone in Exercising Pediatric Populations: A Comprehensive Overview on Endothelial, Antioxidative, Metabolic and Lipoprotein Signaling Molecules

**DOI:** 10.3390/ijms26031027

**Published:** 2025-01-25

**Authors:** Jonas Haferanke, Lisa Baumgartner, Laura Willinger, Renate Oberhoffer-Fritz, Thorsten Schulz

**Affiliations:** Department Health and Sport Sciences, Institute of Preventive Pediatrics, TUM School of Medicine and Health, Technical University of Munich (TUM), 80992 Munich, Germany

**Keywords:** children, adolescents, exercise, vascular adaptation, nitric oxide, endothelin-1, antioxidant enzymes, lipoproteins, leptin, fT3

## Abstract

Vasoactive molecules are central regulators of vascular tone, angiogenesis and inflammation. Key molecular agents include nitric oxide (NO), endothelin-1 (ET-1), prostacyclin, free triiodothyronine (fT3), leptin, low-density lipoprotein (LDL), high-density lipoprotein (HDL), superoxide dismutase (SOD), and glutathione peroxidase (GPX). Dysregulation of these compounds can lead to endothelial dysfunction, an early predictor of atherosclerosis and cardiovascular diseases (CVD). Maintaining endothelial health is thus essential for vascular homeostasis and cardiovascular risk prevention. Regular exercise serves as a vital protective measure against CVD and the risk of cardiovascular conditions. However, young athletes often significantly exceed recommended levels of training load, engaging in highly intensive training that leads to substantial physiological adaptations. Despite this, research on the impact of exercise on vasoactive substances in children and adolescents, particularly young athletes, is limited and inconsistent. Most studies focus on those with pre-existing conditions, like obesity or diabetes mellitus. Existing findings suggest exercise may favorably affect vascular biomarkers in youth, but methodological variations hinder consistent conclusions. This literature review examines 68 studies on the effects of exercise on vascular molecules in children and adolescents, young athletes, and children and adolescents with pre-existing conditions, offering deeper insights into how exercise may influence vascular health at the molecular level.

## 1. Introduction

Vascular active molecules are among the central regulators of vascular function and play a crucial role in a variety of biological processes through their signaling cascades [1,2]. They are significantly involved in the control of vasodilation and vasoconstriction [3,4] but also influence angiogenesis or modulate inflammatory reactions as well as thrombosis formation [5,6]. Among the most important molecules are endothelial agents, such as nitric oxide (NO) [7,8], endothelin-1 (ET-1) [9,10] and prostacyclin [11], metabolic compounds, like free triiodothyronine (fT3) [12] and leptin [13], low-density- (LDL) [14] and high-density-lipoproteins (HDL) [15], and the antioxidant enzymes superoxide dismutase (SOD) [16] and glutathione peroxidase (GPX) [17], as well as various cytokines and chemokines [18,19]. A disturbance of the regulation of these vasoactive substances can promote endothelial dysfunction, an early predictor for the development of atherosclerosis and other cardiovascular diseases (CVD) [20,21]. Therefore, maintaining endothelial health is of central importance for vascular homeostasis and the prevention of cardiovascular risk [22,23]. In the last three decades, research has shown that physical activity affects endothelial health, even in young populations [24,25]. Although studies increasingly focus on different molecular markers of vascular health, investigations into these molecular processes in pediatric populations remain relatively scarce despite a longstanding interest in the topic.

In this context, on the one hand, exercise is one of the main factors causing short- and long-term homeostasis changes in the human body and its subsystems. On the other hand, regular exercise represents an essential protective measure against CVD and offers long-term health benefits by reducing overall mortality rates and decreasing the risk of developing cardiovascular conditions [26,27]. For this reason, the World Health Organization (WHO) recommends that children and adolescents should engage in at least 60 min of moderate to vigorous physical activity daily for preventive purposes [28].

Particular attention should be given to the population of young athletes, as their sporting activity significantly exceeds the WHO recommendations in both volume and intensity [29,30]. Young athletes often engage in several hours per day in intensive training and participation in competition, leading to significant disturbances of the homeostasis and therefore to physiological adaptation [31,32]. However, the influence of these extreme training loads on the regulation of the homeostasis of vasoactive substances is less well understood. There is a possibility that excessive physical strain can lead to both positive and negative changes in vascular function [33,34,35,36].

In adults, the positive influence of exercise on vascular function and the associated modulation of vasoactive substances is well documented. Regular engagement in endurance exercise can increase NO production, leading to improved vasodilation, lowering of blood pressure [37] and, beyond its contribution to endothelial function, an enhancement in exercise performance, particularly for endurance sports [38]. Lipid metabolism also benefits from exercise, as it increases HDL levels and decreases LDL levels, thereby reducing atherogenic risk [39]. Further, the exercise-induced increase in antioxidant capacity by elevated expression of antioxidant enzymes can reduce the amount of oxidative stress [16,40] and pro-inflammatory cytokines are decreased, while anti-inflammatory mechanisms are promoted [41]. Collectively, these modulations contribute to improved endothelial function and a reduction in cardiovascular risk.

By contrast, the research data concerning children and adolescents, particularly healthy young athletes, on the modulation of vascular function and especially the relationship between vascular signaling molecules, such as ET-1, prostacyclin, antioxidant enzymes, leptin, or fT3, and exercise is limited and often inconsistent. Intervention studies in this age group also frequently focus on pediatric populations with already pre-existing conditions, like obesity or diabetes mellitus [42,43,44].

**Figure 1:**
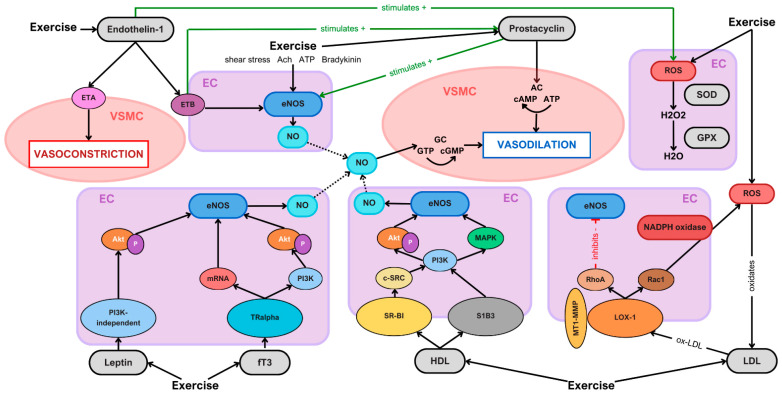
Overview of the signaling network of endothelial, antioxidative, metabolic, and lipoprotein biomarkers and their effect on vascular tone through exercise (EC: endothelial cell; VSMC: vascular smooth muscle cell). HDL and LDL are predominantly synthesized in the liver, leptin is primarily secreted by adipose tissue, and fT3 is produced in the thyroid gland. The bloodstream serves as the central transport medium, delivering these molecules to their respective target sites. Upon binding to their cellular receptors, each biomarker activates its respective pathway, ultimately leading to eNOS activation and subsequent NO release. By contrast, the LDL–LOX-1 signaling pathway leads to an inhibition of eNOS, reducing NO production.

To gain a more comprehensive understanding of how exercise affects vascular health in children and adolescents, the following section provides an overview of the underlying mechanisms of action associated with each vascular signaling molecule. An overview of the molecular pathway network of the selected parameters can also be seen in Figure 1.

## 2. Vascular Signaling Molecules

### 2.1. Endothelial

The vascular endothelium, lining the interior surface of blood vessels, can produce a variety of substances that control vascular constriction and relaxation, such as the vasodilating nitric oxide (NO) and prostacyclin, or the primarily vasoconstricting endothelin-1 (ET-1) [45]. The exercise-induced change in the bioavailability of these substances is a major factor in the regulation of vascular tone [46,47]. Exercise-induced increases in blood flow and shear stress have been demonstrated to enhance endothelial nitric oxide synthase (eNOS) activation, leading to an increased generation of NO [48], and to boost the expression of prostacyclin synthase [49], which is also stimulated by an elevated abundance of NO [50]. Conversely, exercise leads to a decrease in ET-1 production, which is also partially regulated by the presence of NO, acting as an inhibitor of ET-1 synthesis [51]. ET-1 is not stored in granules but is instead synthesized as needed, governed by transcriptional regulation. Therefore, exercise influences these transcriptional mechanisms indirectly, also involving additional factors, such as NFκB, resulting in a multifactorial regulation of ET-1 by exercise [52]. However, the vasoconstrictive effects of ET-1 are dependent on the binding of either one of its two receptor subtypes ET_A_ or ET_B_ [53]. The interaction of ET-1 and the ET_A_ receptor, primarily expressed on smooth muscle cells, induces immediate and potent vasoconstriction. The binding to ET_B_ receptors, primarily expressed on the endothelial cells, leads to the secretion of NO and stimulation of prostacyclin, thereby promoting vascular relaxation. ET-1 released abluminally from the endothelium interacts mainly with ET_A_ receptors, while a small proportion of ET-1 may bind to ET_B_ receptors, limiting the constrictor response by the release of vasodilating factors [54].

### 2.2. Antioxidative

Intense exercise triggers an excessive generation of reactive oxygen species (ROS), shown to be also partially stimulated by an increase in the abundance of ET-1, predominately by the production of superoxide anions (O_2_^−^) [55]. Overproduction of these oxygen radicals is linked to cardiac pathophysiological conditions and molecular-level modifications, such as cardiomyopathy, arrythmia, and damage to mitochondrial DNA. ROS also reduces the bioavailability of nitric oxide (NO) and promotes the oxidation of LDL, main contributing factors to the development of endothelial dysfunction and atherosclerosis [56,57,58,59]. The antioxidant enzymes superoxide dismutase (SOD) and glutathione peroxidase (GPX) play key roles in the protection against exercise-induced oxidative stress as they can eliminate ROS. In adults and trained athletes, especially during endurance exercise, these enzymes can be upregulated in the short term, in an exercise-intensity and duration-dependent manner. SOD converts oxygen radicals into the cell and tissue damaging hydrogen peroxide (H_2_O_2_), which is then subsequently neutralized into water by GPX [40].

### 2.3. Metabolic

Leptin, a mainly metabolically active proteohormone, plays a significant role in regulating food intake and metabolic homeostasis [60]. While primarily secreted by adipose tissue, it can also be released by other tissues, such as skeletal muscle, stomach, and brain [61]. It is rapidly synthesized, with temporally staggered transcriptional regulation influenced by multiple factors, including a wide range of hormones, thereby allowing for quick metabolic adjustments [62]. Regarding vascular health, leptin can exert both proatherogenic and vasoprotective effects, mainly contingent on the balance between physiological and pathological concentrations [63,64]. Abnormally high concentrations of leptin are indirectly linked to several risk factors for atherosclerosis [65]. Leptin receptors are also found directly on the endothelium [66], where leptin stimulates NO synthesis by activating eNOS, resulting in a vasodilating effect [13]. The activation of eNOS by phosphorylation is mediated through a phosphatidylinositol 3-kinase (PI3K)-independent activation of the upstream protein-kinase Akt [67]. Currently, the effect of exercise on leptin remains controversial, as exercise can exert both inhibitory and stimulatory influences on leptin [68], with outcomes highly dependent on intensity for both short- and long-term training, as well as on nutritional status. After a pre-exercise meal, high-intensity exercise leads to an acute reduction in leptin; whereas, under fasting conditions, the degree of leptin reduction depends on exercise volume. Herein, weekly exercise volumes of at least 120 min of high or 180 min of moderate exercise intensity were found to be necessary to significantly decrease basal plasma leptin levels [69]. However, an inverse relationship between physical activity and leptin has been demonstrated even in children far below the activity level of competitive young athletes, where higher physical activity was associated with lower leptin levels [70]. This finding indicates that even minor adjustments in physical activity may affect leptin concentrations.

Thyroid hormones are major metabolic regulators affecting the physiological and pathophysiological processes of the cardiovascular system, including endothelial function, blood pressure, myocardial function, and blood lipids [71]. In elite adult athletes, thyroid hormones are linked to adaptive exercise-induced cardiac remodeling and even small variations in normal range thyroid hormones, like free triiodothyronine (fT3), are associated with adaptations, such as changes in resting and peak heart rate, left ventricular wall thickness, or cardiac mass, across athletes from various sporting disciplines [72]. Thyroid hormone action largely depends on the conversion of the prohormone thyroxine (T4) to the biologically more active form triiodothyronine (T3) by deiodinase (Dio_2_). Most of the T3 is bound to transport proteins and only a small fraction circulates as unbound free T3 (fT3) in the blood stream [73]. fT3 can exert both short and long-term effects on vascular tone by modulating the expression and activity of eNOS [74]. The binding of fT3 on the thyroid hormone receptor alpha (TRalpha) on endothelial cells activates the PI3K/Akt-kinase signaling pathway, leading to the activation of eNOS and thereby enhancing NO synthesis [12]. In the long-term, the binding of fT3 to TRalpha stimulates the expression of eNOS mRNA [75]. Most research on exercise and thyroid function has focused on acute responses to high-intensity exercise in healthy individuals or trained athletes, often showing transient hormonal fluctuations [76,77]. By contrast, the long-term implications of intense training are less well understood, although it has been suggested that prolonged intense exercise may disrupt thyroid function due to the close reliance of training adaptations on these hormones [78,79]. Overall, the evidence regarding the impact of exercise on thyroid hormones is inconclusive. Some studies suggest that exercise can lower fT3 levels by modulating the hypothalamus–pituitary–adipocyte–leptin axis [80], which is associated with a higher risk of CVD [81], while other studies indicate that high-intensity exercise can elevate fT3 concentrations [82]. Whilst the interplay between thyroid function, leptin, and exercise has been recognized [83], it remains inadequately investigated in adolescent athletes.

### 2.4. Lipoproteins

The lipoproteins HDL and LDL play significant roles in cardiovascular health through their synthesis, transport, oxidation, and plasma concentration [84,85]. Higher levels of HDL are associated with a reduced risk of pathological events, while elevated LDL levels correlate with an increased occurrence of such events, like atherosclerosis [86,87]. In terms of vascular health, HDL can activate eNOS, leading to an increased production of NO, resulting in vasodilation [88]. This activation occurs through the binding of HDL to two distinct receptors expressed on endothelial cells [89,90]. Apolipoprotein A-I (apoA-I) binding to the scavenger receptor class B type 1 (SR-B1) stimulates the PI3K/Akt pathway via tyrosine kinase Src, which in turns activates eNOS through phosphorylation [91]. Lysophospholipids of HDL engage the sphingosine-1-phosphate (S1P3) receptor, also activating PI3K, which subsequently stimulates mitogen-activated protein kinase/extracellular-signal-regulated kinase (MAPK/ERK) and Akt, both amplifying eNOS phosphorylation [92].

On the contrary, LDL is associated with inhibiting NO production and the impairment of endothelial function [93,94]. Specifically, the oxidative modification of LDL by ROS into oxidized LDL (oxLDL) triggers various proatherogenic responses [95]. When oxLDL binds to the lectin-like oxLDL receptor (LOX-1), it rapidly activates the GTPases Rhoa and Rac1 via the membrane type-1 matrix metalloproteinase (MT1-MMP), leading to ROS generation via NADPH oxidase activation and downregulation of eNOS [96]. Further, oxLDL disrupts the stimulation of eNOS by the PI3K/Akt pathway by deactivating Akt through LOX-1 activity [97].

Several studies have suggested that exercise training positively influences serum lipid profiles, associated with elevated HDL levels [39,98]. These effects vary depending on factors such as exercise type, intensity, and duration, as well as individual characteristics like body mass, diet and medications [99,100]. Current findings regarding the impact of exercise on LDL levels are somewhat inconsistent [101], nevertheless, research indicates that regular high-intensity exercise is necessary to achieve reductions in LDL levels [102].

A majority of the research on the effects of exercise on the aforementioned vascular molecules has been conducted on adult subjects, with very few studies considering healthy children and adolescents, particularly young athletes. This research gap represents a significant limitation in understanding how vascular health is influenced on a molecular level during sensitive growth periods and crucial developmental stages.

This comprehensive review summarizes the current knowledge about the effects of exercise on selected key vascular biomarker molecules in healthy children and adolescents and young athletes as well as children and adolescents with pre-existing metabolic conditions.

### 2.5. Search Strategy

We conducted a comprehensive literature search in the electronic databases PubMed, Cochrane Library, and Scopus, covering articles published from January 2000 to July 2024. Utilizing a standardized search protocol based on the population, intervention, comparison, outcome and context (PICO) [103] framework, we employed the following combination of search terms: (“physical activity” OR “exercise” OR “athlete”) AND (“children” OR “adolescents”) AND (“vascular function” OR “endothelial function” OR “arterial stiffness” OR “arterial pressure” OR “vasoconstriction” OR “vasodilation” OR “blood flow”) AND (“nitric oxide” OR “endothelin-1” OR “prostacyclin” OR “superoxide dismutase” OR “glutathione peroxidase” OR “leptin” OR “fT3” OR “HDL” OR “LDL”). Medical Subject Headings (MeSH) terms and relevant filters were applied to refine the search parameters, including publication date range, human subjects, and specific age groups (children: 6–12 years; adolescents: 13–18 years). These filters were appropriately adjusted as necessary to enhance the specificity and sensitivity of the search.

A majority of the identified studies address only a single aspect or component of the search terms. As a result, the findings were systematically categorized into four distinct sections to comprehensively present the various dimensions of exercise and vascular health in children and adolescents and to facilitate a thorough analysis and synthesis of the evidence related to different molecular biomarkers.

## 3. Impact of Exercise on Vascular Biomarkers

### 3.1. Endothelial

We identified twelve published studies (Table 1) that examined the effect of exercise on the endothelial biomarkers NO, ET-1 and prostacyclin in children and adolescents. Of these, two were interventional studies, nine were interventional studies with a control group, and one was a cross-sectional study with a control group. Regarding the study populations, four of the studies were conducted on healthy children and healthy young athletes, while the remaining eight studies investigated children and adolescents with pre-existing conditions.

In healthy children, Souza et al. [104] demonstrated that engaging in moderate to vigorous physical activity for 10 weeks, at 75–85% of heart rate reserve (HRR) for four sessions of 45 min per week, resulted in a significant increase in NO levels compared to baseline. Additionally, Stergioulas et al. [105] showed that performing aerobic cycle ergometer exercise for 60 min, four times a week at 80% of maximal physical working capacity, led to a significant elevation in plasma prostacyclin levels after eight weeks. Similarly, male adolescent wrestlers significantly enhanced their NO levels after undergoing a 90-min mixed aerobic endurance, strength, and sport-specific skill training [106]. However, in a study by Djordjevic et al. [107], NO levels remained unchanged between male handball players and non-athlete controls after a maximal progressive cycle-ergometer test until exhaustion. In children with pre-existing conditions, ET-1 levels notably decreased after 12 weeks of combined exercise training at 60–70% of heart rate reserve (HRR) three times per week for 60 min in obese girls [111], as well as in obese adolescents following a supervised soccer training program for 12 weeks [43]. Asthmatic children who underwent a cycling exercise intervention twice a week at 80% of their submaximal heart rate (HR), along with pharmacological treatment, also experienced a significant decrease in ET-1 levels [112], accompanied by a notable decrease in NO levels [110]. Conversely, ET-1 levels remained unaffected in obese adolescents following a six-week program of either moderate or high-intensity interval training at 65–70% or 90–95% of maximal heart rate (HRmax), respectively [114]. Similarly, after 12 weeks of 60 min treadmill and resistance band exercises at 40–70% of HRR in female obese adolescents, ET-1 levels remained unchanged [109], however, NO levels significantly increased following the intervention. Park et al. [108] also observed increased NO concentrations in obese children participating in a 12-week after-school combined aerobic and resistance exercise program at 50–70% of HRR and 60% of the 1-repetition maximum (1 RM). Moreover, a significant increase in the ratio of NO/ET-1 was noted in obese adolescents who underwent a combination of aerobic and resistance exercise program for six weeks at 60–70% of HRmax and 60–70% of 1 RM [113].

In summary, of the twelve identified studies, four reported elevated levels of nitric oxide (NO) and one indicated an increase in prostacyclin following exercise, both of which are favorable for improved vascular dilation. A reduction in NO was observed in only one study, while decreases in the vasoconstrictor endothelin-1 (ET-1) were reported in three studies, also supporting improved vascular stiffness. Notably, one study demonstrated that exercise enhanced the ratio of NO/ET-1. Additionally, two studies found no impact of exercise on ET-1 levels, and one study showed no effect on NO levels.

### 3.2. Antioxidative

We identified nine studies (Table 2) that explored the relationship between exercise and the antioxidative markers SOD and GPX among children and adolescents. Of these, two were intervention studies, two were cross-sectional studies, one was a cross-sectional study with a control group, and four were intervention studies that included control groups. Regarding the study populations, two of the studies were conducted with healthy children, four studies included healthy young athletes, and three studies had children and adolescents with pre-existing conditions as subjects.

Gonenc et al. [115] demonstrated that four weeks of swimming exercise, seven days per week for 120 min each, led to a significant increase in SOD levels but had no impact on GPX levels in untrained healthy children. Conversely, Paltoglou et al. [116] revealed a significant increase in GPX levels in pre- and early pubertal boys following an aerobic exercise session on a cycle ergometer at 70% VO_2_max until exhaustion. Among healthy young athletes, male handball players exhibited notably higher SOD levels compared to non-athlete controls after maximal progressive cycle ergometer exercise [107]. Likewise, male soccer players showed significantly elevated SOD levels after six months of soccer-specific skill and endurance training totaling at least 12 h per week [117]. By contrast, adolescent female gymnasts experienced a significant decrease in SOD levels compared to non-athlete controls after a longitudinal 3-year observation period with a training volume of at least 10 h per week [119], while GPX levels increased significantly during the same period compared to controls. Tong et al. [118] examined experienced adolescent runners in a pre- and post-one-year training time trial of a 21 km endurance run, involving 180–240 min of training six times a week, covering 60–80 km of running distance. After both test runs, SOD levels decreased significantly. After an eight-week cycle ergometer training at 50% above resting heart rate combined with pharmacological treatment, both SOD and GPX levels significantly increased in asthmatic children [112], as well as in physically active boys with type 1 diabetes mellitus following a 12-week treadmill training three times per week at 45–55% heart rate reserve [44]. Additionally, a single bout of exercise at 70% VO_2_maxuntil exhaustion on a cycle ergometer led to significant increases in GPX levels in obese pre- and early pubertal boys [116].

Taken together, five studies each reported increased levels of superoxide dismutase (SOD) and glutathione peroxidase (GPX) following exercise interventions, suggesting improved mitigation of oxidative stress, thereby improving vascular protection, while two studies indicated decreased SOD levels, and one study found no effect on GPX levels in response to exercise.

### 3.3. Metabolic

Sixteen studies (Table 3) were analyzed to investigate the impact of exercise on metabolic markers. Of these, one was a cross-sectional study, three were cross-sectional studies with control groups, twelve were intervention studies, of which eight studies had control groups. In terms of study populations, one study focused on healthy children, two examined healthy young athletes, and thirteen involved children and adolescents with pre-existing conditions. No studies met our inclusion criteria regarding the effect of exercise on the thyroid hormone fT3; therefore, the subsequent analyses exclusively address leptin as a metabolic biomarker.

Leptin levels did not show any significant changes in healthy schoolboys immediately following a 30-min acute cycle ergometer exercise at 95% of their individual ventilatory threshold [120]. However, there were notable reductions in leptin levels observed in pre-pubertal female swimmers after a maximal aerobic endurance test [121], as well as in young female athletes engaged in gymnastics, ballet, and acrobatics training for 10–12 h per week over a minimum of five years, compared to non-athlete controls [122]. Several studies have examined the impact of exercise on leptin levels in obese children and adolescents. Lopes et al. [42] found a significant decrease in leptin levels in overweight girls after a four-week combined aerobic and resistance training program, a result similarly observed by Li et al. [126] in obese girls undergoing 16 weeks of aerobic exercise at 65–70% HRmax. Obese boys also exhibited decreasing leptin levels following aerobic exercise, with significant reductions noted after 12 weeks of thrice-weekly sessions [123] and six months of either aerobic exercise or soccer training [124]. Concurrent training in obese boys for 12 weeks likewise led to significant reductions in leptin levels [125]. Additionally, Siegrist et al. [128] and Kelishadi et al. [127] both observed reductions in leptin levels in response to aerobic exercise in obese children after six weeks and six months of training, respectively. Racil et al. [130] reported significant decreases in leptin in obese adolescents undergoing high-intensity interval training (HIIT) and combined HIIT plus plyometric exercises. Elloumi et al. [131] observed a significant decline in leptin in two experimental groups of obese adolescents performing either aerobic exercise or aerobic exercise combined with caloric energy restriction. Kamal et al. [129] found a significant reduction in leptin in obese children following a 12-week aerobic exercise intervention at 60–65% HRR. However, Souza et al. [132] did not report any changes in leptin in obese children after a maximal progressive aerobic endurance test, nor did Vasconcellos et al. [43] observe changes in leptin levels in obese adolescents after a 12-week soccer training program. Similarly, Lau et al. [133] found that leptin levels remained unaffected by six weeks of resistance exercise at 85% of 1 RM in overweight adolescents.

To summarize, among the sixteen identified studies, a total of twelve reported reductions in leptin levels following an exercise intervention, while four indicated that exercise had no impact on leptin concentrations. Additionally, elevated levels of leptin may favorably influence vascular relaxation through its stimulatory effect on nitric oxide (NO) secretion. This suggests that, although the majority of studies observed a decrease in leptin with exercise, instances of increased leptin could potentially enhance endothelial function and promote vasodilation, thereby contributing positively to vascular health.

### 3.4. Lipoproteins

We identified 31 intervention studies (Table 4) that investigated the effect of exercise on the lipoproteins HDL and LDL in children and adolescents, of which 24 studies had a control group. In terms of the study populations, eight of the studies were conducted on healthy children and adolescents, one study involved healthy young athletes and twenty-two studies investigated subjects with pre-existing conditions.

In healthy children undergoing aerobic exercise on a cycle ergometer four times per week for eight weeks at 80% of their physical working capacity, HDL levels significantly increased after the intervention [105]. Likewise, healthy adolescents engaged in endurance exercises of varying intensity five times per week for five weeks exhibited elevations in HDL levels, while LDL levels remained unchanged [134]. Contrarily, healthy adolescents performing three weekly sessions of 4–6 20-m sprint exercises for seven weeks experienced a significant decrease in LDL levels post-intervention, with no change in HDL levels [135]. Similarly, Rosenkranz et al. [136] demonstrated that a high-intensity aerobic exercise intervention twice a week for eight weeks reduced LDL levels but had no effect on HDL levels. However, no significant changes in either HDL or LDL levels were observed in healthy children after 12 weeks of aerobic exercise [138] and 12 weeks of aerobic cycle ergometer exercise at 80% HRmax [137], as well as after 20 weeks of aerobic exercise at 75–80% HRmax in healthy girls [139]. Ghorbanian et al. [140] also found that rope training for eight weeks in healthy male adolescents did not alter LDL levels post-intervention. In young healthy swimmers and soccer players, 12 weeks of specific sports training led to a significant increase in HDL levels compared to a sedentary control group in both swimmers and soccer players, while a significant reduction in LDL levels was only observed in the soccer group [141].

The predominant conditions observed in studies exploring the impact of exercise on lipoprotein levels among children and adolescents are obesity and overweight. Karacabey et al. [123], Zorba et al. [144], and Kamal et al. [129] found that aerobic walking and jogging exercises over 12 weeks at 60–65% HRR resulted in significant reductions in LDL levels and significant increases in HDL levels in obese boys and children. Kamal et al. [129] also noted a marked rise in HDL concentration in obese children with metabolic syndrome following aerobic exercise. In children with type 1 diabetes mellitus, Aouadi et al. [145] observed a significant decrease in LDL and an elevation of HDL after six months of aerobic exercise at 50–65% HRmax. Lee et al. [148] reported a reduction in LDL concentration after ten weeks of aerobic exercise in obese children, while a combination of aerobic and resistance training yielded lower LDL and higher HDL levels. Seabra et al. [124] found that soccer training, but not aerobic exercise, over six months at 70–80% HRmax led to lower LDL levels and higher HDL levels. Woo et al. [147] demonstrated that a combination of aerobic exercise and dietary intervention led to decreased LDL levels but unchanged HDL levels after six weeks in overweight children but increased HDL levels along with reduced LDL after 12 months. Racil et al. [146] observed significant decreases in LDL levels after 12 weeks of moderate-intensity interval training and HIIT, with a simultaneous significant increase in HDL in both intervention groups among obese adolescent girls. Kovács et al. [149] observed significant decreases in LDL levels but unaltered HDL concentrations following five weeks of aerobic exercise in obese children, while Meyer et al. [155] found similar results after six months of aerobic exercise in obese adolescents. The studies by Chae et al. [150], on obese children undertaking 12 weeks of combined aerobic and resistance training, and Zehsaz et al. [154], on obese male children after 16 weeks of aerobic and resistance exercise, also demonstrated reductions in LDL levels. Roberts et al. [151], Korsten-Reck et al. [152], and Kelishadi et al. [153] reported significant decreases in LDL levels but unchanged HDL levels in obese children following a combined aerobic exercise and dietary intervention. Sung et al. [158] noted a decrease in LDL levels in obese children after a six-week program comprising combined aerobic and resistance training alongside a dietary intervention. By contrast, Kelly et al. [142] found significantly elevated HDL levels but no change in LDL levels in overweight children and adolescents after eight weeks of aerobic cycle ergometer exercise, while Ribeiro et al. [143] reported similar results in obese children after four months of a combined aerobic exercise and dietary intervention. Farpour-Lambert et al. [156] and Sun et al. [157] observed simultaneous decreases in both LDL and HDL levels in obese children and adolescents, respectively, following aerobic and strengthening exercises. Kelishadi et al. [127], Migueles et al. [159], and Benson et al. [160] reported no changes in either LDL or HDL levels in obese children following various exercise interventions. Similarly, Wong et al. [161] found no changes in lipoprotein concentrations in obese adolescents after 12 weeks of a combined aerobic and resistance exercise program.

To sum up, of the 33 studies reviewed, 13 indicated an elevation in HDL levels following exercise, while 2 studies observed decreases, and 17 studies found no significant effect on HDL. In terms of LDL, 23 studies reported reductions post-exercise intervention, while 11 studies observed no change in LDL concentrations. Elevated HDL levels enhance vascular health by providing atheroprotective effects while reduced LDL levels lower the risk of arterial cholesterol deposition and atherosclerosis.

## 4. Conclusions and Future Directions

The studies reviewed indicate that exercise can modulate a broad spectrum of endothelial, antioxidative, metabolic, and lipoprotein biomarkers associated with vascular health. Numerous investigations demonstrate that exercise enhances the balance of vascular regulators—elevating levels of vasodilatory compounds, like nitric oxide (NO) and prostacyclin, while reducing vasoconstrictive agents, such as endothelin-1 (ET-1), thus favorably altering the overall dilator-to-constrictor ratio. Additionally, exercise enhances the presence of the antioxidant enzymes SOD and GPX across different exercise interventions and study populations. Multiple studies have also shown that exercise lowers leptin concentrations, reflecting favorable metabolic adaptations. However, data on the influence of exercise on the thyroid hormone free triiodothyronine (fT3) remain unavailable, as no studies met the inclusion criteria for investigating thyroid hormones in pediatric populations in relation to exercise, given that children and adolescents undergo age-specific developmental changes, e.g., with TSH levels typically decreasing over the course of growth before stabilizing in adulthood [162]. Such age-related variations in thyroid function and hormone dynamics underscore the need for focused research in this demographic. Regarding lipoproteins, exercise tends to decrease LDL levels and elevate HDL levels, effects particularly evident in studies involving children and adolescents with pre-existing conditions, such as overweight and obesity.

These findings in children and adolescents align with similar research in adults and adult athletes. Maeda et al. [163] demonstrated that both resistance and aerobic exercise lower plasma levels of the endothelial biomarker ET-1 in healthy adults, and Hansen et al. [49] showed that exercise enhances the capacity to produce the vasodilator prostacyclin in sedentary men. Interestingly, Zoladz et al. [164] observed responders and non-responders in exercise-induced prostacyclin increases in physically active men. A meta-analysis by Arefirad et al. [165] indicated that NO levels increase with exercise, regardless of duration or type of training. By contrast, increases in the antioxidative enzymes SOD and GPX were found to be specific to the sport discipline- and intensity of activity, as shown by Dékány et al. [166], which showed results similar to the results of Souissi et al. [167] in adult endurance athletes, which showed that SOD levels increase after a continuous running exercise but not after an intermittent running protocol, while GPX levels remain unchanged in both groups. Regarding the metabolic marker leptin, a meta-analysis by Fontana et al. [69] revealed that both short- and long-term training reduces leptin levels, with effects of acute exercise depending on pre-exercise nutritional status. Concerning lipoproteins, a systematic review by Mann et al. [102] concluded that regular exercise increases HDL levels in adults in a linear dose-response relationship between activity levels and HDL levels, and that a higher exercise intensity is required to elicit reductions in LDL levels. A significantly larger number of included studies focus on exercise-related effects on lipoproteins in pediatric populations compared to the other biomarkers. This discrepancy likely reflects several factors. HDL and LDL are widely recognized risk markers for cardiovascular disease [168], have well-established and standardized measurement protocols, and are thus straightforward to link to exercise interventions. In addition, dyslipidemia can manifest during childhood [169], prompting investigations to check whether early exercise regimens may mitigate these risk factors. By contrast, studies exploring possible mechanistic molecular or metabolic pathways, e.g., via release and change of blood levels of hormonal mediators, antioxidative enzymes, or endothelial factors, often require more specialized assays and lack uniform methodologies, contributing to the relative scarcity of work in these areas.

Although most identified studies indicate that exercise may positively influence vascular biomarkers in children and adolescents, the overall evidence remains inconsistent, likely due to methodological heterogeneity and variation in measurement techniques. These methodological discrepancies substantially hinder the comparability of findings across studies. For instance, blood sampling schedules may vary by the time of the day or in the time passed after the intervention, and the age ranges of children and adolescents can differ considerably. Study populations also include healthy untrained individuals (13 studies), young athletes (9 studies) and children with pre-existing conditions like obesity or diabetes (46 studies). In addition, variation in exercise type, duration, frequency, and intensity introduces further complexity, making it particularly challenging to distinguish between short- and long-term adaptations. Notably, research specifically examining young competitive athletes under the age of 18—a population of particular importance for understanding vascular adaptations—is extremely limited. To address this gap, future investigations with clearly defined methodologies should focus on this demographic. Additionally, the scarcity of data on long-term adaptations beyond 12 months highlights the need for extended longitudinal studies of young athletes, particularly during their sensitive phases of growth and development. From a broader perspective, possible aspects of future research should include integrating additional biomarkers to gain a deeper understanding of how exercise influences vascular health and the underlying mechanisms in children and adolescents [170]. Building on this, advanced profiling techniques, spanning genomics, transcriptomics, proteomics, and metabolomics, could provide a comprehensive biomarker profile that captures the full spectrum of molecular changes induced by exercise [171]. Investigations could also explore the interplay of genetic predisposition, nutritional factors, and training regimens to better understand individualized responses and tailor exercise prescriptions. Such efforts would not only clarify the mechanisms behind vascular adaptations but guide evidence-based recommendations for pediatric exercise programs aimed at optimizing long-term cardiovascular health.

## Figures and Tables

**Table 1:** Overview of studies that examine the effect of exercise on the endothelial biomarkers NO, ET-1, and prostacyclin.

Healthy Individuals						
Reference	N	Age	Population/Source/ Testing	Intervention	Duration/ Intensity	Result
Souza et al. (2019) [104]	40	7–11	Healthy children / Blood plasma / Nitrate/Nitrite	Moderate to vigorous physical activity	45 min, 4x per week, for 10 weeks / 75–85% HRR	**NO** ↑ (*p* < 0.001)
Stergioulas et al. (2006) ^a^ [105]	38	10–14	Healthy children / Urine / Prostanoid metabolite	Aerobic exercise on cycle ergometer	60 min, 4x per week, for 8 weeks / 80% of physical working capacity	**Prostacyclin** ↑ (74.3 ± 6.5 to 115.9 ± 11.2 pg/ng, *p* < 0.001)
**Healthy** **athletes**						
Hamurcu et al. (2010) [106]	18	13.89 ± 0.95	Male adolescent wrestlers / Blood serum / Nitrate/Nitrite	Mixed aerobic endurance, wrestling skill and strength training	90 min, 6x per week	**NO** ↑ (11.79 ± 3.73 to 15.94 ± 5.09 μM, *p* = 0.002)
Djordjevic et al. (2011) ^b^ [107]	33	16–19	Male handball players / Blood plasma / Nitrate/Nitrite	Maximal progressive testing on cycle ergometer	Start at 2 W/kg, increase every 3 min for 50 W; 60 rpm, until oxygen consumption plateau	**NO** ↔ (athletes vs. non-athlete control)
**Pre-existing condition**						
Park et al. (2012) [108]	29	12–13	Obese children / Blood plasma / Nitrate/Nitrite	After school combined aerobic and resistance exercise	80 min, 3x per week, for 12 weeks / Aerobic: 50–70%HRR Resistance: 60% 1 RM, 8–12 repetitions	**NO** ↑ (8.1 ± 0.6 to 10.6 ± 1.0 μM, *p* < 0.001)
Wong et al. (2018) [109]	30	15.2 ± 1.2	Female obese adolescents / Blood plasma / Nitrate/Nitrite (NO), Protein level (ET1)	Treadmill and resistance band exercises	60 min, 3x per week, for 12 weeks / Increasing from 40–50%HRR to 60–70% HRR	**NO** ↑ (53.7 ± 4.9 to 57.7 ± 4.1 μM, *p* = 0.03) **ET1 ↔**
Onur et al. (2011) ^c^ [110]	15	8–13	Asthmatic children / Blood serum / Nitrate/Nitrite	Cycle ergometer exercise + pharmacological treatment	60 min, 2x per week, for 8 weeks / 80% of submaximal HR	**NO** ↓ (11.84 ± 2.24 to 9.09 ± 1.96 μmol/L, *p* = 0.001)
Son et al. (2017) [111]	40	14–16	Obese girls / Blood plasma / Protein level	Combined exercise training	60 min, 3x per week For 12 weeks / 60–70% HRR, RPE 15–16	**ET1** ↓ (14.35 ± 1.76 μmol/mL, *p* < 0.05)
Gunay et al. (2012) ^c^ [112]	30	8–13	Asthmatic children / Blood serum / Protein level	Cycle ergometer exercise + pharmacological treatment	60 min, 2x per week, for 8 weeks / 80% of submaximal HR	**ET1** ↓ (26.5 ± 3.6 to 21.3 ± 2.4 pg/mL, *p* < 0.001)
Vasconcellos et al. (2016) ^d^ [43]	10	12–17	Obese adolescents / Blood plasma / Protein level	Soccer training program	60 min, 3x per week, for 12 weeks	**ET1** ↓ (2.1 ± 0.5 to 1.7 ± 0.5 pg/mL, *p* < 0.042)
Donghui et al. (2019) [113]	57	12–18	Obese adolescents / Blood serum / Protein level	Exercise + dietary restriction	Aerobic: 80 min, 5x per week, For 6 weeks / 60–70% max HR Resistance: 60 min, 2x per week, For 6 weeks / 60–70% 1 RM, 8.12 repetitions	Ratio **NO/ET1** ↑ (1.73 ± 0.51 vs. 2.72 ± 0.92, *p* < 0.01)
Starkoff et al. (2015) [114]	27	14.7 ± 1.5	Obese adolescents / Blood serum / Protein level	High-intensity interval training or moderate-intensity training	HIIT: 30 min, 3x per week, for 6 weeks / 90–95% HRmax MOD: 30 min, 3x per week, for 6 weeks / 65–70% HRmax	**ET1** ↔

HR: heart rate; HRR: heart rate reserve; HRmax: maximum heart rate; 1 RM: 1-repetition maximum; W: watt; W/kg: watt per kilogram; rpm: rounds per minute; RPE: rate of perceived exertion; HIIT: high-intensity interval training; MOD: moderate-intensity interval training. ↑: substance level increased; ↓: substance level decreased; ↔: no change. Superscripted lowercase letters (^x^) next to study citations indicate that those studies originate from the same research group.

**Table 2:** Overview of studies that examine the effect of exercise on the antioxidative biomarkers SOD and GPX.

Healthy Individuals						
Reference	N	Age	Population/Source/ Testing	Intervention	Duration/ Intensity	Result
Gonenc et al. (2000) [115]	12	6–11	Untrained healthy children / Isolated RBC / Enzyme activity	Swimming exercise	120 min, 7x per week, for 4 weeks	**SOD** ↑ (581.1 ± 146.2 to 791.1 ± 221.9 U/gHb, *p* < 0.01) **GPX↔**
Paltoglou et al. (2019) ^e^ [116]	65	10.32 ± 0.24 (pre-pubertal) 11.53 ± 0.22 (early pubertal)	Normal weight pre- and early pubertal boys / Whole blood / Enzyme activity	Aerobic exercise bout on cycle ergometer	70% VO_2_max until exhaustion	**GPX** ↑ prepubertal (3350.85 ± 68.69 to 4202 ± 60.02 U/L, *p* < 0.05) early pubertal (3329.40 ± 189.35 to 4261.40 ± 234.3 U/L, *p* < 0.05)
**Healthy athletes**						
Djordjevic et al. (2011) ^b^ [107]	33	16–19	Male handball players / Isolated RBC / Enzyme activity	Maximal progressive testing on cycle ergometer	Start at 2 W/kg, increase every 3 min for 50 W; 60 rpm, until oxygen consumption plateau	**SOD** ↑ (2175.52 ± 362.0 vs. 1172.16 ± 747.40 U/g, athletes vs. non-athlete control, *p* < 0.05)
Zivkovic et al. (2013) [117]	6	12–13	Male soccer players / Isolated RBC / Enzyme activity	6-month training program, soccer-specific skill and endurance training	75–90 min, at least 12 h per week, for 6 months	**SOD** ↑ (*p* < 0.05)
Tong et al. (2013) [118]	10	14–17	Experienced adolescent runners / Blood serum / Enzyme activity	21 km endurance run time trial pre- and post-1-year training	180–240 min per day, 6.5x per week / 7–21 km per day, 60–80 km per week	**SOD** ↓ (pre) (69.2 ± 12.3 to 63.4 ± 15.6 U/mL, *p* < 0.05) **SOD** ↓ (post) (67.0 ± 13.5 to 60.9 ± 13.9 U/mL, *p* < 0.05)
Alshammari et al. (2010) [119]	38	8–17	Adolescent female gymnasts / Blood serum / Enzyme activity	3 year-longitudinal observation	Training volume >10 h/week	**GPX** ↑ (156.92 ± 11.07 vs. 125.14 ± 8.79 U/mL; athletes vs. non-athlete control, *p* < 0.05) **SOD** ↓ (7.23 ± 0.41 vs. 8.57 ± 0.385 U/mL; athletes vs. non-athlete control *p* < 0.05)
**Pre-existing condition**						
Paltoglou et al. (2019) ^e^ [116]	27	10.43 ± 0.38 (pre-pubertal) 11.71 ± 0.33 (early pubertal)	Obese pre- and early pubertal boys / Whole blood / Enzyme activity	Aerobic exercise bout on cycle ergometer	70% VO_2_max until exhaustion	**GPX** ↑ prepubertal (2804.11 ± 143.94 to 3671.44 ± 161.67) U/L, *p* < 0.05) early pubertal (3227.33 ± 97.72 to 4065.22 ± 132.31 U/L, *p* < 0.05)
Onur et al. (2011) ^c^ [110]	30	8–13	Asthmatic children / Blood plasma / Enzyme activity	Cycle ergometer exercise + pharmacological treatment	60 min, 2x per week, for 8 weeks / HR at 50% above resting HR	**SOD** ↑ (5.49 ± 3.80 to 13.03 ± 5.54 U/mL, *p* = 0.001) **GPX** ↑ (160.13 ± 56.03 to 242.06 ± 81.94 U/L, *p* = 0.003)
Woo et al. (2010) [44]	10	10–14	Physically active boys with T1DM / Blood plasma / Enzyme activity	Treadmill exercise	3x per week, for 12 weeks / 45–55% HRR	**SOD** ↑ (*p* < 0.05) **GPX** ↑ (*p* < 0.05)

VO_2_max: maximal oxygen consumption; HR: heart rate; HRR: heart rate reserve; W: watt; W/kg: watt per kilogram; rpm: rounds per minute; T1DM: type 1 diabetes mellitus; RBC: red blood cells. ↑: substance level increased; ↓: substance level decreased; ↔: no change. Superscripted lowercase letters (^x^) next to study citations indicate that those studies originate from the same research group.

**Table 3:** Overview of studies that examine the effect of exercise on the metabolic biomarkers leptin and fT3.

Healthy Individuals						
Reference	N	Age	Population/Source/ Testing	Intervention	Duration/ Intensity	Result
Pomerants et al. (2006) [120]	60	10–18	Healthy schoolboys / Blood serum / Protein level	Acute cycle ergometer exercise	30 min at 95% of IVT	**Leptin** ↔
**Healthy athletes**						
Güllü et al. (2020) [121]	16	9.88 ± 1.41	Pre-pubertal swimmer Girls / Blood serum / Protein level	Stepwise maximal aerobic endurance test	Progressive protocol until exhaustion	**Leptin** ↓ (11.67 ng/mL to 8.53 ng/mL, *p* < 0.004)
Jürimäe et al. (2017) [122]	60	10–12	Girl athletes from gymnastics, ballet and acrobatics / Blood plasma / Protein level	Testing of nationally competing athletes compared to non-athlete control	Athletes trained: 5–7x per week, 10–12 h per week for at least 5 years	**Leptin** ↓ 2.4 ± 1.1 ng/mL vs. 7.6 ± 4.2 ng/mL, *p* < 0.05, athletes vs-non-athlete control)
**Pre-existing condition**						
Lopes et al. (2016) [42]	17	13–17	Overweight girls / Blood serum / Protein level	Combined aerobic and resistance training	60 min, 3x per week, for 4 weeks / Resistance: 6–10 repetitions on machines Aerobic: 50–85% VO_2_peak running on track	**Leptin** ↓ (*p* < 0.05)
Karacabey et al. (2009) ^f^ [123]	20	10–12	Obese boys / Blood serum / Protein level	Aerobic exercise walking/jogging	30–60 min, 3x per week, for 12 weeks / 60–65% HRR	**Leptin** ↓ (23.3 ± 9.9 to 16.7 ± 9.6 ng/mL, *p* < 0.001)
Seabra et al. (2016) ^d^ [124]	58	8–12	Obese boys / Blood plasma / Protein level	Aerobic exercise group and soccer group	Both groups: 60–90 min, 3x per week, for 6 months / 70–80% HRmax	**Leptin** ↓ (Soccer: 21.6 + 16.1 to 16.1 + 13.3 ng/mL, *p* < 0.05) (Aerobic: 27.1 + 21.0 to 20.9 + 19.8 ng/mL, *p* < 0.05)
Fazelifar et al. (2013) [125]	12	11–13	Obese boys / Blood serum / Protein level	Concurrent training	3x per week, for 12 weeks	**Leptin** ↓ (*p* < 0.05)
Li et al. (2022) [126]	16	12.04 ± 0.96	Obese girls / Blood serum / Protein level	Aerobic exercise	60 min, 4x per week, for 16 weeks / 65–70% HRmax	**Leptin** ↓ (22.05 ± 4.80 to 19.17 ± 4.51 ug/L, *p* < 0.01)
Kelishadi et al. (2008) ^g^ [127]	45	7.7 ± 1.2	Obese children / Blood serum / Protein level	Aerobic exercise	40 min, 5x per week, for 6 months	**Leptin** ↓ (*p* < 0.05)
Siegrist et al. (2013) [128]	402	13.9 + 2.3	Obese children / Blood serum / Protein level	Aerobic exercise group sports/walking	16 h/week, for 6 weeks	**Leptin** ↓ (39.5 + 23.7 to 18.6 + 14.0 ng/mL, *p* < 0.001)
Kamal et al. (2012) ^h^ [129]	44	8–12	Obese children with/without metabolic syndrome / Blood plasma / Protein level	Aerobic exercise walking/jogging	30–60 min, 3x per week, for 12 weeks / 60–65%HRR	**Leptin** ↓ (for w/o MS, *p* < 0.05)
Racil et al. (2016) ^i^ [130]	49	16.6 ± 1.3	Obese adolescents / Blood plasma / Protein level	HIIT group and plyometric exercise + HIIT group	HIIT: 3x per week, for 12 weeks / 6–8 bouts of 30 s runs at 100% velocity HIIT + P: 3x per week, for 12 weeks / HIIT program + 3x 2 min plyometric exercises	**Leptin** ↓ (HIIT: 20.2 ± 2.6 to 17.3 ± 1.8 ng/mL, *p* = 0.033) (HIIT + P: 17.6 ± 2.3 to 13.5 ± 2.0 ng/mL, *p* = 0.019)
Elloumi et al. (2009) [131]	7	13.2 ± 0.9	Obese adolescent boys / Blood plasma / Protein level	Exercise group and energy restriction + exercise group	Exercise: 90 min, 4x per week, for 8 weeks / HR corresponding to LipoMax Exercise + Diet: identical exercise + 500 kcal below initial dietary record	**Leptin** ↓ (Exercise: *p* < 0.05) (Energy Restriction + Exercise: *p* < 0.01)
Vasconcellos et al. (2016) ^d^ [43]	10	12–17	Obese adolescents / Blood plasma / Protein level	Soccer training program	60 min, 3x per week, for 12 weeks	**Leptin** ↔
Souza et al. (2004) [132]	40	6–11	Obese children / Blood serum / Protein level	Stepwise maximal aerobic endurance test	Progressive protocol until exhaustion	**Leptin** ↔
Lau et al. (2010) [133]	18	12.45 ± 1.77	Overweight adolescents / Blood serum / Protein level	Resistance exercise	60 min, 3x per week, for 6 weeks / Up to 85% of 1 RM	**Leptin** ↔

IVT: individual ventilatory threshold; VO_2_peak: peak oxygen uptake at the end of exercise; HR: heart rate; HRR: heart rate reserve; HRmax: maximum heart rate; 1 RM: 1-repetition maximum; HIIT: high-intensity interval training. ↑: substance level increased; ↓: substance level decreased; ↔: no change. Superscripted lowercase letters (^x^) next to study citations indicate that those studies originate from the same research group.

**Table 4:** Overview of studies that examine the effect of exercise on the lipoprotein markers HDL and LDL.

Healthy Athletes						
Reference	N	Age	Population/Source/ Testing	Intervention	Duration/ Intensity	Result
Eliakim et al. (2000) [134]	20	15–17	Healthy adolescents / Blood serum / Protein level	Endurance exercise	12–150 min, 5x per week, for 5 weeks / Duration and intensity varied	**HDL** ↑ (32.6 ± 1.4 to 36.8 ± 1.6 mg/dL, *p* < 0.05) **LDL** ↔
Stergioulas et al. (2006) ^a^ [105]	38	10–14	Healthy children / Blood serum / Protein level	Aerobic exercise on cycle ergometer	60 min, 4x per week, for 8 weeks / 80% of physical working capacity	**HDL** ↑ (1.24 + 0.17 to 1.45 + 0.31 mmol/L, *p* < 0.01)
Buchan et al. (2013) [135]	42	16.7 ± 0.6	Healthy adolescents / Blood plasma / Protein level	Sprint running	3x per week, for 7 weeks / 4–6 repeats of max. 20 m sprint	**LDL↓** (2.5 + 1.5 to 1.5 + 1.0 mmol/L, *p* < 0.019) **HDL** ↔
Rosenkranz et al. (2012) [136]	16	7–12	Healthy children / Whole blood / Protein level	High intensity aerobic exercise	30 min, 2x per week, for 8 weeks / 5 × 20 s Running intervals at 100–130% max. aerobic speed	**LDL↓** (97.5 + 18.8 to 62.7 + 20.2 mg/dL, *p* < 0.05) **HDL** ↔
Tolfrey et al. (2004) [137]	34	10.6 ± 0.6	Healthy children / Blood plasma / Protein level	Aerobic stationary cycling exercise	Individual duration, 3x per week, for 12 weeks / 80% of HRmax	**LDL/HDL** ↔
Balas-Nakash et al. (2010) [138]	319	8–12	Healthy children / Blood plasma / Protein level	Aerobic exercise	40 min, 5x per week, for 12 weeks	**LDL/HDL** ↔
Stoedefalke et al. (2000) [139]	20	13–14	Healthy girls / Blood serum / Protein level	Aerobic exercise	20 min, 3x per week, for 20 weeks / 75–85% HRmax	**LDL/HDL** ↔
Ghorbanian et al. (2013) [140]	30	14–17	Healthy male adolescents / Blood plasma / Protein level	Rope training	40 min, 4x per week, for 8 weeks	**LDL** ↔
**Healthy athletes**						
Koozehchian et al. (2014) [141]	27	11.81 ± 1.38	Swimmers and soccer players / Blood plasma / Protein level	Swimming training or soccer training	60 min, 3x per week, for 12 weeks	**LDL↓** (*p* < 0.01, only for soccer) **HDL** ↑ (*p* < 0.05, for swimming and soccer)
**Pre-existing condition**						
Kelly et al. (2004) [142]	25	10.9 ± 0.4	Overweight children and adolescents / Blood serum / Protein level	Aerobic exercise cycle ergometer	30–50 min, 4x per week, for 8 weeks / 50–80% VO_2_peak	**HDL** ↑ (1.02 ± 0.03 to 1.10 ± 0.04 mmol/L, *p* < 0.05) **LDL** ↔
Ribeiro et al. (2005) [143]	21	10 ± 0.2	Obese children / Blood serum / Protein level	Diet and aerobic exercise	60 min, 3x per week, for 4 months / HR levels correspond to anaerobic threshold up to 10% below respiratory compensation point	**HDL** ↑ (39 ± 0.8 to 44 ± 0.5 mg/dL, *p* < 0.05) **LDL** ↔
Karacabey et al. (2009) ^f^ [123]	20	10–12	Obese boys / Blood serum / Protein level	Aerobic exercise walking/jogging	30–60 min, 3x per week, for 12 weeks / 60–65% HRR	**LDL↓** (87.2 ± 9.4 to 67.5 ± 9.4 mg/dL, *p* < 0.001) **HDL** ↑ (51.9 ± 7.5 to 59.0 ± 7.5 mg/dL, *p* < 0.001)
Seabra et al. (2016) ^d^ [124]	58	8–12 y	Obese boys / Blood plasma / Protein level	Aerobic exercise group and soccer group	Both groups:60–90 min, 3x per week, for 6 months / 70–80% HRmax	**LDL↓** only soccer (104.3 + 42.2 to 90.9 + 32.3 mg/dL, *p* < 0.05) **HDL** ↑ only soccer (53.6 + 9.5 to 57.7 + 12.1 mg/dL, *p* < 0.05)
Zorba et al. (2011) ^f^ [144]	20	11 ± 1.0	Obese children / Blood serum / Protein level	Aerobic exercise walking/jogging	20–45 min, 3x per week, for 12 weeks / 60–65% HRmax	**LDL** ↓ (87.2 + 9.4 to 67.5 + 9.4 mg/dL, *p* < 0.001) **HDL** ↑ (51.9 + 7.5 to 59 + 7.5 mg/dL, *p* < 0.001)
Kamal et al. (2012) ^h^ [129]	44	8–12	Obese children with/without metabolic syndrome / Blood serum / Protein level	Aerobic exercise walking/jogging	30–60 min, 3x per week, for 12 weeks / 60–65%HRR	**LDL** ↓ (w/o MS: 107 ± 10.2 to 99.4 ± 15.4 mg/dL, *p* < 0.05) (w MS: non-significant changes) **HDL** ↑ (w/o MS: 45.8 ± 5.9 to 49.3 ± 6.5 mg/dL, *p* < 0.05) (w MS: 32 ± 2.7 to 43.4 ± 6.2 mg/dL, *p* < 0.05)
Aouadi et al. (2011) [145]	11	13.5 ± 0.8	Children with T1DM / Blood serum / Protein level	Aerobic exercise	60 min, 4x per week, for 6 months / 50–65% HRmax	**LDL** ↓ (81.6 + 11.8 to 69.2 + 8.5 mg/dL, *p* < 0.01) **HDL** ↑ (56.7 + 7.2 to 68.8 + 5.7 mg/dL, *p* < 0.01)
Racil et al. (2013) ^i^ [146]	22	15.9 ± 0.3	Obese adolescent girls / Blood plasma / Protein level	High-intensity interval training or Moderate-intensity interval training	3x per week, for 12 weeks / HIIT: 100–110% max aerobic speed MIIT: 70–80% max aerobic speed	**LDL** ↓ (HIIT: 2.49 ± 0.32 to 2.18 ± 0.4 mmol/L, *p* < 0.01 MIIT: 2.77 ± 0.3 to 2.55 ± 0.32 mmol/L, *p* < 0.05) **HDL** ↑ (HIIT: 1.02 ± 0.06 to 1.08 ± 0.08 mmol/L, *p* < 0.05 MIIT: 1.01 ± 0.08 to 1.09 ± 0.07 mmol/L, *p* < 0.05)
Woo et al. (2004) ^j^ [147]	41	9–12	Overweight children / Blood serum / Protein level	Diet + aerobic exercise	75 min, 2x per week, for 6 weeks / 60–70% HRmax	**LDL** ↓ (2.9 + 0.9 to 2.6 + 0.8 mmol/L, *p* < 0.002) **HDL** ↔
Woo et al. (2004) ^j^ [147]	22	9–12	Overweight children / Blood serum / Protein level	Diet + aerobic exercise	75 min, 2x per week, for 12 months / 60–70% HRmax	**LDL** ↓ (3.0 + 0.9 to 2.7 + 1.0 mmol/L, *p* < 0.05) **HDL** ↑ (1.2 + 0.3 to 1.4 + 0.3 mmol/L, *p* < 0.01)
Lee et al. (2010) ^k^ [148]	16	12–14	Obese children / Blood serum / Protein level	Aerobic exercise	60 min, 3x per week, for 10 weeks / 70–90% HRmax	**LDL** ↓ (116.07 ± 28.08 mg/dL vs. 103.73 ± 27.48 mg/d, *p* < 0.05)
Lee et al. (2010) ^k^ [148]	20	12–14	Obese children / Blood serum / Protein level	Aerobic and resistance exercise	60 min, 3x per week, for 10 weeks / Resistance:70–80% max. strength Aerobic: 70–90% HRmax	**LDL** ↓ (115.42 ± 14.13 mg/dL vs. 105.68 ± 16.43 mg/dL, *p* < 0.05) **HDL** ↑ (45.26 ± 7.07 mg/dL vs. 49.47 ± 9.13 mg/dL, *p* < 0.05)
Kovács et al. (2009) [149]	38	6.5–12.5	Obese children / Blood plasma / Protein level	Aerobic exercise	60 min, 3x per week, for 5 weeks / Working HR 120–185 bpm	**LDL** ↓ (2.4 ± 0.6 vs. 1.9 ± 0.6 mM/L, *p* < 0.0001) **HDL** ↔
Chae et al. (2010) [150]	19	9–15	Obese children / Blood plasma / Protein level	Intensive aerobic and resistance exercise	90 min, 2x per week, for 12 weeks	**LDL** ↓ (101.3 ± 6.3 to 90.2 ± 6.5 mg/dL, *p* < 0.05) **HDL** ↔
Roberts et al. (2013) [151]	19	8–17	Obese children / Blood serum / Protein level	Diet + aerobic exercise	120–150 min, 7x per week, for 2 weeks	**LDL** ↓ (95.0 ± 6.1 to 71.8 ± 5.0 mg/dL, *p* < 0.01) **HDL** ↔
Korsten-Reck et al. (2005) [152]	461	10.5	Obese children / Blood plasma / Protein level	Diet + aerobic exercise	60 min, 3x per week, for 8 months	**LDL** ↓ (106.0 + 28.9 to 100.2 + 25.8 mg/dL, *p* < 0.001) **HDL** ↔
Kelishadi et al. (2008) ^g^ [153]	35	12–18	Obese children / Blood serum / Protein level	Diet and exercise	60 min, 3x per week, for 6 weeks / Moderate to vigorous intensity	**LDL** ↓ (3.1 ± 0.5 to 2.7 ± 0.4 mmol/L, *p* < 0.02) **HDL** ↔
Zehsaz et al. (2016) [154]	16	9–12	Obese male children / Blood serum / Protein level	Aerobic and resistance training	Aerobic: 30 min, 2x per week, for 16 weeks / 55–75% HRmax Resistance: 55 min, 2x per week, for 16 weeks / 70% 1 RM	**LDL** ↓ (87.2 ± 9.4 to 72.4 ± 9.5 mg/dL, *p* < 0.001) **HDL** ↔
Meyer et al. (2006) [155]	33	14.7 ± 2.2	Obese adolescents / Blood plasma / Protein level	Aerobic exercise	60–90 min, 3x per week, for 6 months	**LDL** ↓ (2.71 + 0.7 to 2.57 + 0.66 mmol/L, *p* = 0.025) **HDL** ↔
Farpour-Lambert et al. (2009) [156]	22	8.9 ± 1.5	Obese children / Blood plasma / Protein level	Aerobic and strengthening exercise	Aerobic: 30–40 min, 3x per week, for 12 weeks Strength: 20 min, 3x per week, for 12 weeks	**LDL** ↓ (*p* < 0.05) **HDL** ↓ (*p* < 0.01)
Sun et al. (2011) [157]	25	13.6 ± 0.7	Obese adolescents / Blood serum / Protein level	Aerobic exercise	60 min, 4x per week, 10 weeks / 40–60% VO_2_max	**LDL** ↓ (2.6 ± 0.6 to 2.3 ± 0.5 mmol/L, *p* < 0.01) **HDL** ↓ (1.2 ± 0.2 to 1.1 ± 0.2 mmol/L, *p* < 0.01)
Sung et al. (2002) [158]	41	8–11	Obese children / Blood serum / Protein level	Diet + aerobic and resistance exercise	75 min/session, for 6 weeks / 60–70% HRmax	**LDL** ↓ (2.9 ± 0.8 to 2.6 ± 0.8 mmol/L, *p* < 0.05)
Migueles et al. (2023) [159]	47	8–11	Obese children / Blood serum / Protein level	Aerobic and resistance exercise	60 min aerobic, 30 min resistance, 3x per week, for 20 weeks	**LDL/HDL** ↔
Benson et al. (2008) [160]	32	12.2 ± 1.3	Obese and overweight children / Blood serum / Protein level	Progressive resistance training	2x per week, for 8 weeks / 80% 1 RM	**LDL/HDL** ↔
Wong et al. (2008) [161]	12	13–14	Obese adolescents / Blood serum / Protein level	Aerobic and resistance exercise	2x per week, for 12 weeks / 65–85% HRmax	**LDL/HDL** ↔
Kelishadi et al. (2008) ^g^ [127]	45	7.7 ± 1.2	Obese children / Blood serum / Protein level	Aerobic exercise	40 min, 5x per week, for 6 months	**LDL/HDL** ↔

HR: heart rate; HRR: heart rate reserve; HRmax: maximum heart rate; bpm: beats per minute; VO_2_max: maximum oxygen consumption; VO_2_peak: peak oxygen uptake at the end of exercise; HIIT: high-intensity interval training; MIIT: moderate-intensity interval training; 1 RM: 1-repetition maximum; T1DM: type 1 diabetes mellitus; MS: metabolic syndrome. ↑: substance level increased; ↓: substance level decreased; ↔: no change. Superscripted lowercase letters (^x^) next to study citations indicate that those studies originate from the same research group.

## Data Availability

Not applicable.

## Abbreviations

*NO*	nitric oxide
*ET-1*	endothelin-1
*fT3*	free triiodothyronine
*SOD*	superoxide dismutase
*GPX*	glutathione peroxidase
*HDL*	High-Density Lipoprotein
*LDL*	Low-Density Lipoprotein
*oxLDL*	oxidized Low-Density Lipoprotein
*CVD*	cardiovascular disease
*WHO*	World Health Organization
*eNOS*	endothelial nitric oxide synthase
*ET_A_*	endothelin receptor subtype A
*ET_B_*	endothelin receptor subtype B
*ROS*	reactive oxygen species
*PI3K*	phosphatidylinositol 3-kinase
*T4*	thyroxine
*T3*	triiodothyronine
*TRalpha*	thyroid hormone receptor alpha
*SR-B1*	scavenger receptor class B type 1
*Src*	tyrosine protein kinase Src
*Akt*	Protein Kinase B
*S1P3*	sphingosine-1-phosphate receptor
*MAPK/ERK*	mitogen-activated protein kinase/extracellular-signal-regulated kinase
*LOX-1*	Lectin-like oxLDL receptor
*MT1-MMP*	membrane type-1 matrix metalloproteinase
*NFκB*	Nuclear factor kappa B
